# Influence of Incident Wavelength and Detector Material Selection on Fluorescence in the Application of Raman Spectroscopy to a Fungal Fermentation Process

**DOI:** 10.3390/bioengineering5040079

**Published:** 2018-09-25

**Authors:** Stephen Goldrick, David Lovett, Gary Montague, Barry Lennox

**Affiliations:** 1 Biopharmaceutical Bioprocess Technology Centre, Merz Court, Newcastle University, Newcastle-upon-Tyne NE1 7RU, UK; s.goldrick@ucl.ac.uk; 2 Perceptive Engineering Limited, Vanguard House, Keckwick Lane, Daresbury, Cheshire WA4 4AB, UK; dlovett@perceptiveapc.com; 3 School of Science, Engineering & Design, Teeside University, Tees Valley TS1 3BX, UK; G.Montague@tees.ac.uk; 4 Control Systems Group, School of Electrical and Electronic Engineering, University of Manchester, Manchester M13 9PL, UK

**Keywords:** Raman spectroscopy, fluorescence, fermentation monitoring, PLS modelling, critical process parameters, critical quality attributes

## Abstract

Raman spectroscopy is a novel tool used in the on-line monitoring and control of bioprocesses, offering both quantitative and qualitative determination of key process variables through spectroscopic analysis. However, the wide-spread application of Raman spectroscopy analysers to industrial fermentation processes has been hindered by problems related to the high background fluorescence signal associated with the analysis of biological samples. To address this issue, we investigated the influence of fluorescence on the spectra collected from two Raman spectroscopic devices with different wavelengths and detectors in the analysis of the critical process parameters (CPPs) and critical quality attributes (CQAs) of a fungal fermentation process. The spectra collected using a Raman analyser with the shorter wavelength (903 nm) and a charged coupled device detector (CCD) was corrupted by high fluorescence and was therefore unusable in the prediction of these CPPs and CQAs. In contrast, the spectra collected using a Raman analyser with the longer wavelength (993 nm) and an indium gallium arsenide (InGaAs) detector was only moderately affected by fluorescence and enabled the generation of accurate estimates of the fermentation’s critical variables. This novel work is the first direct comparison of two different Raman spectroscopy probes on the same process highlighting the significant detrimental effect caused by high fluorescence on spectra recorded throughout fermentation runs. Furthermore, this paper demonstrates the importance of correctly selecting both the incident wavelength and detector material type of the Raman spectroscopy devices to ensure corrupting fluorescence is minimised during bioprocess monitoring applications.

## 1. Introduction

Raman spectroscopy is a non-invasive, non-destructive spectroscopic technique that exploits molecular vibrations for the qualitative and quantitative analysis of molecules [1]. It has broad applications in biology and chemistry and has been applied in environmental and industrial applications [2]. Interest in this form of spectral analyser from the biotechnology industry has gained momentum in recent years [3], prompted by the release of the Process Analytical Technology (PAT) initiative by the FDA in 2004 [4]. The primary advantages of Raman spectroscopy as a PAT analyser relevant to bioprocesses include, small sample volume requirement, no sample preparation, little interference from water in the analysis of aqueous samples, ability to analyse through glass or plastic and high specificity for a wide of nutrients and products [5,6]. Recent demonstrations of Raman spectroscopy applied to bioprocesses have included real-time monitoring of nutrient concentrations and viable cell densities in mammalian cell culture runs [7,8], ethanol production in *Saccharomyces cerevisiae* fermentations [9,10] and nutrient and phenylalanine concentrations in an *Escherichia coli* fermentation [11]. More advanced demonstrations include the on-line monitoring of a recombinant antibody titre during a mammalian cell cultivation [12], in addition to the ability of Raman spectroscopy to monitor complex post-translational modifications as shown by Li et al. [13] in the real-time monitoring of glycosylation during monoclonal antibody production.

It is clear that Raman spectroscopy will play a pivotal role in the real-time monitoring and control of bioprocesses. However, a major hurdle hindering the wide-spread adoption of these process analysers relates to the high fluorescence observed during the analysis of biological molecules which often overlay the important Raman scattering bonds, diminishing the ability to estimate the material of interest [14,15]. There are various different methods to alleviate or suppress fluorescence in the analysis of biological materials. Photo-bleaching has been demonstrated to reduce the recorded fluorescence in the analysis of bone tissue through prolonged exposure of the sample to intense excitation from the laser source decomposing fluorophores responsible for sample fluoresence [16]. Adjustments to the confocal set-up has also been reported to reduce fluorescence by reducing its depth of focus which effectively reduces the path length reducing the detected fluorescence resultant from outside of the laser focus [17]. A technique known as shifted excitation Raman difference spectroscopy (SERDS) involving the collection and subtraction of two Raman spectra in succession at slightly different laser wavelengths was also demonstrated to eliminate fluorescence during the analysis of biological samples [17,18]. This technique creates a derivative-like spectrum with the background fluorescence signal eliminated, enabling better resolution of the important Raman features [19]. Furthermore a technique known as time-gated Raman spectroscopy can reduce fluorescence by exploiting the differing time scales between Raman scattering and fluorescence absorbance. Whereas Raman scattering is completed almost instantaneous (<1 picosecond) and fluorescence emission takes up 100–1000 times longer (nanosecond range). Time-gated Raman spectroscopy works by illuminating a sample for a very short time using a laser pulse. Provided the detection system is gated as to only detect those photons scattered or emitted during the first few picoseconds only the important Raman photons will be recorded while rejecting the majority of the unwanted fluorescence photons [20,21]. In addition to these techniques the choice of the excitation wavelength of the Raman device can significantly impact the level of observed fluorescence for the majority of samples based on the inverse relationship between the excitation wavelength of the Raman device and the probability of sample fluorescence [2]. For example, Ultra-Violet Raman spectroscopy enables better noise-to-signal ratios due to the lower wavelength and also can reduce the fluorescent interference as most species do not fluorescence below an excitation band of 260 nm [15,22]. The detector material of the device can also be highly influential on observed fluorescence, however, little research has been reported on the importance of this selection criteria in the application of Raman spectroscopy to fermentation monitoring.

To address this issue and advance the use of this technology in fermentation applications, two Raman spectroscopic analysers were implemented on a highly fluorescence fungal fermentation process. One Raman analyser had an incident wavelength of 903 nm and used a silicon-based charged couple device (CCD) detector and the second device had a 993 nm wavelength with an indium gallium arsenide (InGaAs) array detector. Both analysers were implemented on a similar small-scale fungal fermentation process with the objective of estimating the critical process parameters (CPPs) and critical quality attributes (CQAs) of the fermentation. These have been previously identified for this process as the glucose and active pharmaceutical ingredient (API) concentration, respectively. The spectral data collected using the Raman device with the shorter wavelength and CCD detector was found to be significantly corrupted by a high background fluorescence signal in contrast to the 993 nm Raman device with the InGaAs detector which was only moderately affected by fluorescence. The spectra collected from both analysers was correlated with the off-line concentrations of both variables using partial least squares (PLS) modelling. Only the regression models generated using the spectra recorded on the 993 nm device enabled accurate predictions of both the glucose and API concentration. To the best of the authors’ knowledge, this is the first direct comparison of two Raman spectroscopy devices with different incident wavelengths and detector material to monitor the same fermentation process. This work highlights the need to better understand the fundamental principles of fluorescence on recorded Raman spectra and demonstrates the importance of correct probe selection in future applications of this novel technology to the biotechnology sector.

## 2. Experimental Section

### 2.1. Microorganism and Media

A proprietary fungus supplied by Pfizer was used to inoculate both fermentations that was propagated from the same thawed culture stock supplemented with a proprietary nutrient feed. The fungus produces a high concentration of a commercially available antibiotic, referenced as the active pharmaceutical ingredient (API) concentration.

### 2.2. Bioreactor Conditions

Two fed-batch fungal fermentations (referred to as Fermentation A and Fermentation B) were performed in a 5 L bioreactor with a working volume of approximately 3.6 L. Each bioreactor was set to have identical operating conditions, both equipped with thermometers, dissolved oxygen and pH probes. The temperatures of the reactors were kept at 28 ${}^{\circ }$C using an external cooling jacket. The pH of the culture was maintained at 6.2 by the addition of an acid/base solution using a proportional integral derivative (PID) controller. Mixing was accomplished using a standard Ruston impeller operating at a fixed RPM. The air flow rate was fixed to its upper limit for both fermentations. Off-line measurements from Fermentation A were recorded once a day and for Fermentation B were recorded three times a day. Glucose concentration was measured using an off-line analyser and the concentration of the active pharmaceutical ingredient (API) was determined through high pressure liquid chromatography (HPLC). Throughout the batch, glucose was controlled through Bolus glucose additions. Anti-foam additions were added as required. Specific details regarding the microorganism, media composition and vendor selections have been omitted for reasons of confidentiality.

### 2.3. Raman Spectroscopy Devices

In Fermentation A, a 993 nm Raman spectroscopy device with an indium gallium arsenide (InGaAs) detector array with a spectral range of 200–2400 cm${}^{-1}$ and a resolution of 3 cm${}^{-1}$ was implemented. In Fermentation B the laser wavelength of the Raman device was equal to 903 nm with a spectral range of 200–2400 cm${}^{-1}$ and a resolution of 3 cm${}^{-1}$ using a silicon-based CCD detector. Each device was connected to a portable computer that collected the spectra on-line and allowed for both the integration time and number of averages to be manually adjusted throughout each fermentation. Before use, each Raman device was calibrated to ensure each pixel number was correlated to the correct wavenumber in the spectrograph. The calibration was performed by analysing the Raman spectra of a known material and comparing the wavenumbers of the main peaks in the spectra to ensure they corresponded to the known wavenumbers of the sample. Additional calibration samples were made up to help identify the peaks of interest, these involved analysing the Raman spectra collected from aqueous samples spiked with glucose (20 g L${}^{-1}$) and API (6 g L${}^{-1}$) additions.

### 2.4. Raman Spectra Preprocessing and Wavelength Selection

The spectra collected by each device was combined with the off-line glucose and API measurements and was used to generate two PLS models. In Fermentation A, ten off-line glucose samples were recorded and 18 off-line samples for Fermentation B. For each fermentation eight off-line samples of the API concentration were recorded. The off-line glucose samples were interpolated using a cubic spline approximation and a 30-min sampling rate resulting in 522 and 475 sample points for Fermentation A and B, respectively. The off-line API concentrations were interpolated in a similar fashion resulting in approximately 360 sample points. The 30-min sampling rate was chosen to match the sampling frequency of each Raman device that was set up to produce a single spectra every 30 min through adjustment of the number of averages and integration times. The preprocessing of the spectra utilised the de-spiking algorithm outlined in Mori et al. [23] and was baseline corrected as shown in Eilers and Boelens [24]. The spectra were further preprocessed by calculating the first derivative of the spectra using a Savitzky-Golay filter with a width of 15 and a polynomial order of 5, similar to the preprocessing method outlined in Bocklitz et al. [6]. The preprocessed spectral data ($X_{spec}$) and the corresponding off-line glucose (**Y**${}_{Gluc}$) and off-line API concentrations (**Y**${}_{API}$) were divided up to ensure each calibration data set adequately described the concentration ranges of both the glucose and API in the validation data sets. Thus, the first 75 h of each fermentation were used for the calibration data sets for the glucose measurements with the remaining used to validate the model. The calibration data used for the prediction of the API concentration used the first six off-line measurements, additionally five interpolated data points around each of the off-line API concentrations were included in the calibration data set for both Fermentation A and B, consisting of a total of 30 data points. The validation data set consisted of the data between these calibration data points and the remaining data after the sixth off-line API concentration value.

**Figure A1:**
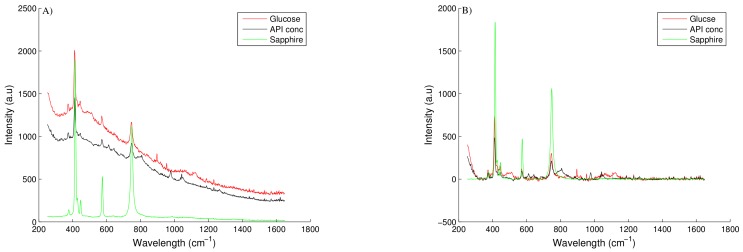
Raman spectra of calibration samples highlighting glucose, API and sapphire (background spectra) spectrographs, with (**A**) highlighting the raw spectra and (**B**) highlighting the baseline corrected spectra.

The wavelengths associated with the glucose were identified through the analysis of the aqueous calibration samples spiked with high concentrations of glucose (20 g L${}^{-1}$) and were taken as: 366:372 456:476 477:486 891:897 898:919 1589:1595 cm${}^{-1}$. The spectra collected during the analysis of the calibration samples are available in Appendix A shown in Figure A1. The PLS model for the API concentration was generated in a similar manner, taking the wavelengths as 720:732 786:793 800:806 cm${}^{-1}$. The optimum number of components for each PLS model were chosen based on the root mean square error of correlation (RMSEC) and root mean square error of prediction (RMSEP) as defined in Equation (6).

### 2.5. Partial Least Model Generation

The PLS model implemented the non-linear iterative partial least squares (NIPALS) algorithm as outlined in detail by Wold et al. [25]. The preprocessed spectral data ($X_{spec}$) was first decomposed in to *R* latent variables, generating a matrix of scores, **T**, and loadings, **P** with **E** as the residuals. The off-line concentration of the glucose concentration ($Y_{Gluc}$) was decomposed in a similar fashion generating a matrix of scores, **U**, and loadings, **Q** with **F** as the residuals, defined below as:

(1) $$X_{spec}={\mathrm{TP}}^{\prime }+E$$

(2) $$Y_{Gluc}={\mathrm{UQ}}^{\prime }+F$$

A vector of inner-relationships **B** is generated that relates scores of the **X** block to the **Y** block as:

(3) $$B=X_{spec}^{T}T{\left(X_{spec}^{T}X_{spec}\right)}^{-1}$$

The PLS model works iteratively for each latent variable and upon convergence a matrix of regression coefficients β can be generated as follows:

(4) $$\beta =W{\left(P^{T}W\right)}^{-1}\mathrm{diag}\left(B\right)$$

The cumulative sum of the regression coefficients predicts the response variable ($\hat{Y_{Gluc}}$) from the **X** block taking *R* latent variables:

(5) $$\hat{Y_{Gluc}}=X_{spec}\sum_{r=1}^{R}\beta$$

Similar procedure was undertaken for predictions of the active pharmaceutical ingredient ($Y_{API}$).

### 2.6. Validation of PLS Model

To select the number of latent variables to choose in the PLS model, the prediction error of the model was calculated. The error related to the calibration data set was calculated using the root mean square error of calibration (RMSEC) and for the validation data set the root mean square error of prediction (RMSEP) was used. These functions were calculated as described in [26]:

(6) $$\begin{array}{cc} \mathrm{RMSEC} & ={\left[\frac{1}{n}\sum_{i=1}^{n}{\left(y_{i}-\hat{y_{i}}\right)}^{2}\right]}^{0.5}\\ \mathrm{RMSEP} & ={\left[\frac{1}{p}\sum_{i=1}^{p}{\left(y_{i}-\hat{y_{i}}\right)}^{2}\right]}^{0.5} \end{array}$$

where:
*n*:calibration samples*p*:validation samples$y_{i}$:*i*th calibration sample$\hat{y_{i}}$:*i*th validation sample

### 2.7. Raman Spectroscopic Fundamentals

**Figure 1:**
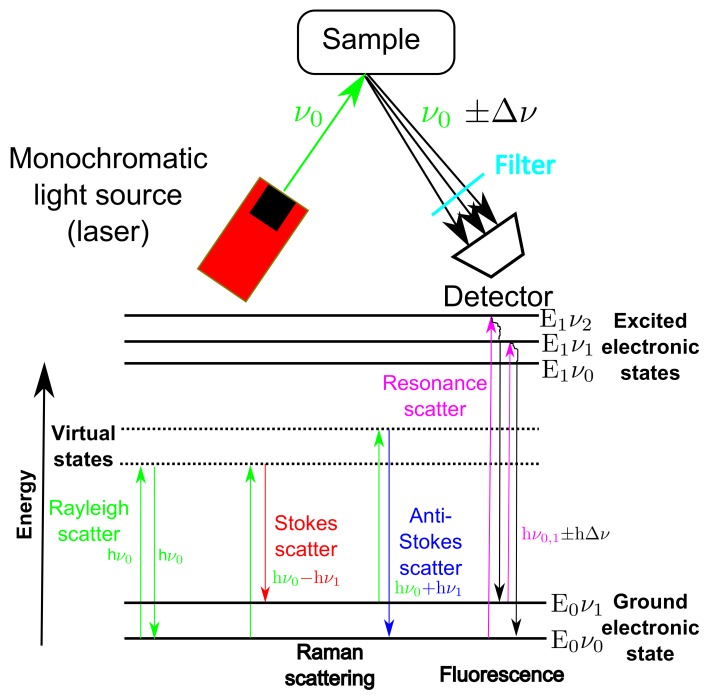
Schematic of the fundamental principles of Raman spectroscopy, highlighting the main scattering and fluorescence excitations of a sample after it is excited using a monochromatic light source of frequency equal to $\nu_{0}$.

The fundamental principles of Raman spectroscopy are outlined in Figure 1. The process involves illuminating a sample using a monochromatic light source of fixed frequency equal to $\nu_{0}$ and analysing the scattered light that is recorded using a detector. The energy of the light source is given by E = h$\nu_{0}$ with h equal to Plank’s constant and $\nu_{0}$ equal to its frequency. The interaction of the light with the sample can result in small frequency shifts (Δν) and a resultant energy deviation. The interactions of the light with the sample result in various scattering and absorbance phenomena as highlighted in Figure 1.

Rayleigh scattering occurs when the light interacts with the molecules of the sample and the net exchange of energy is zero i.e., energy of the incident light (h$\nu_{0}$) is equal to the energy of the scattered light. Conversely, if the sample gains energy from the light and is shifted up one vibrational state then the frequency of the scattered light will be lower than the incident beam i.e., energy of scattered light (h$\nu_{0}$ − hΔν) will be less than the energy of incident light (h$\nu_{0}$), referred to as Stokes scatter. If the interaction causes the sample to lose energy then the frequency of the scattered light will be higher than the incident light i.e., the energy of the reflected beam (h$\nu_{0}$+hΔν) will be greater than the energy of the incident beam (h$\nu_{0}$), this is known as anti-stokes scatter. It is the Stokes shifted scatter that is usually measured by Raman spectroscopic analysers and referred to as Raman shift or Raman scatter and often measured in terms of wavenumber in units of cm${}^{-1}$, typical ranging from 200 to about 3000 cm${}^{-1}$.

Both of these scattering phenomena result in the excitation of electrons of the sample to virtual states which are lower in energy than an excited electronic transition state (E’). The net energy deviation results in characteristic peaks in the resultant spectra. The positions of these peaks are defined by the molecular structure of the sample and its chemical environment, allowing Raman spectroscopy to be used for chemical identification and classification. Furthermore, the peak heights (or areas) of the spectrum are assumed linearly proportional to the molecular concentration and consequently can be used to monitor the CPPs or CQAs of bioprocesses, provided the Raman analyser can detect the material of interest [27].

The intensity of Raman scattering ($I_{\mathrm{Raman}}$) is very weak and is often difficult to detect, the weakness of this signal is the primary limitation of Raman spectroscopy. This is evident by comparing the intensity of Raman scattering to that of the source ($I_{\mathrm{Source}}$) and the signal received due to Rayleigh scattering ($I_{\mathrm{Rayleigh}}$) which was defined by [28] to be in the following range:

(7) $$I_{\mathrm{Raman}}\approx 10^{-4}I_{\mathrm{Rayleigh}}\approx 10^{-8}I_{\mathrm{Source}}$$

It is therefore necessary to filter out the Rayleigh scattered light in order to detect the weak Raman scattering effect [29].

In competition with the weak Raman scattering is fluorescence which is a non-scattering process that occurs when the incident beam absorbs some energy from the light source and temporarily excites the electrons with enough energy to be transferred up to a higher quantum state (E’). There are multiple higher quantum states that the exited electrons can obtain and this is dependent on the energy and wavelength of the external light source. The electrons in their excited state are unstable and as they return to their respective ground state they release light with energy equal to $h\nu_{0,1}$ ± hΔν as highlighted in Figure 1. The other main difference between fluorescence and Raman scattering is the time-scale involved in each process, with the fluorescence process taking in the region of nanoseconds ($10^{-9}$ s) compared to the Raman scattering process which is much quicker and is completed in picoseconds ($10^{-12}$ s) [28].

Molecules that are susceptible to this fluorescence process when excited by visible, ultra-violet or near-infrared light are known as fluorophores or fluorescence molecules, these are typically polyaromatic hydrocarbons or heterocycle molecules with several π bonds. In fermentation monitoring, there are many known and unknown biological compounds that fluoresce. Typically, proteins, enzymes, vitamins and primary and secondary metabolites from microbial growth have this property [5], however culture fluorescence can also be related to culture conditions including cell density, viscosity and product concentration [30]. Unfortunately in fermentation monitoring the onset of fluorescence is a major problem with only one in every $10^{4}$ scattered photons related to Raman scattering, even small levels of fluorescence can mask out the signal rendering the analysis very difficult or redundant.

## 3. Results and Discussion

### 3.1. Fluorescence Observations

**Figure 2:**
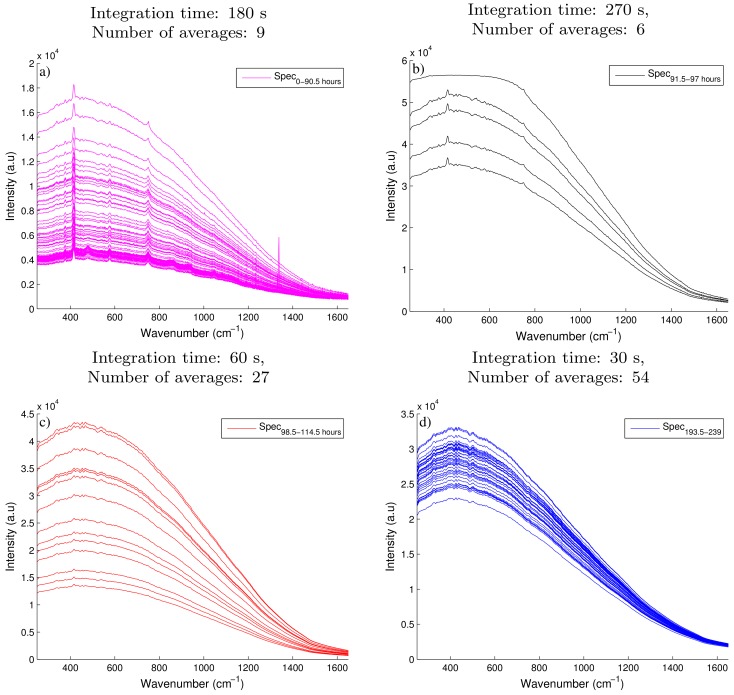
A subset of the raw spectra recorded by the 903 nm Raman spectroscopic device collected at different tuning parameters with (**a**) recorded using an integration time of 180 s and taking 9 averages over the time course of the fermentation from 0–90.5 h; (**b**) had an integration time of 270 s and 6 averages over the time course of 91.5–97 h; (**c**) had an integration time of 60 s and 27 averages over the time course of 98.5–114.5 h and (**d**) was recorded using an integration time of 30 s and 54 averages over the time course of 193.5–239 h.

**Figure 3:**
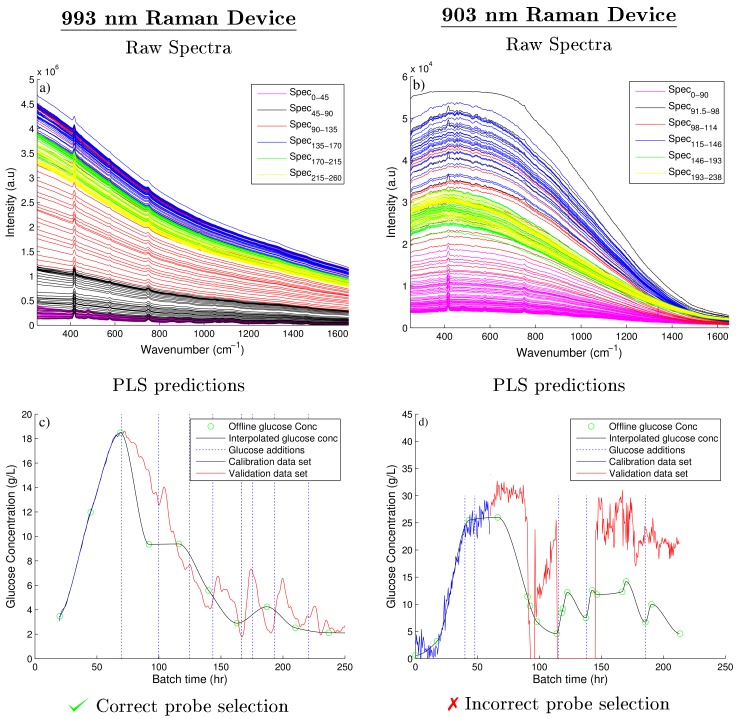
Raw spectra collected from the 993 and 903 nm Raman spectroscopic devices, shown in (**a**,**b**) respectively. The corresponding PLS model predictions compared against the on-line glucose concentrations are shown in (**c**,**d**), respectively

The spectra collected from two Raman spectroscopic devices were analysed to estimate the glucose and API concentration, previously identified as the primary CPP and CQA on this small-scale fungal fermentation. The spectra collected on the 993 nm Raman device was moderately influenced by fluorescence in comparison to the spectra recorded by the 903 nm Raman device which was significantly effected by fluorescence. Figure 2a highlights the large baseline shift which was mainly attributed to fluorescence of the fermentation broth recorded on the 903 nm Raman device during the first 90.5 h of the fermentation. A fourfold increase is observed in intensity as the baseline spectra shifts from 0.4 × 10${}^{4}$ a.u to nearly 1.6 × 10${}^{4}$ a.u. Although this large increase is not atypical, Cannizzaro et al. [31] observed a similar increase in baseline shift during the first 90 h of a fed-batch process monitoring a *Phaffia rhodozyma*. However, some of the important Raman peaks observed in Figure 2a are shown to be dwarfed by the strong background fluorescence signal in comparison to the initial spectra recorded. These spectra were recorded using an integration time of 180 s taken 9 averages. In an attempt to improve the resolution of these Raman peaks and improve the signal to noise ratio, the tuning parameters of the Raman 903 nm device were adjusted throughout the batch as defined in Table 1. Although it is not recommended to adjust the tuning parameters of a Raman device during a fermentation due to the complications in the generation of the subsequent regression model. To ensure the important Raman peaks are not obscured by the prominent broad fluorescence signals in-process changes are often necessary. Shih and Smith [32] previously demonstrated that an increase in the integration time improved the resolution of the glucose peaks recorded on the spectra collected using a 785 nm Raman device during an ethanol producing fermentation. In the experiments performed here a similar increase in the intensity of the spectra was observed after the integration time was increased to 270 s and the number of averages reduced to 6, as seen in Figure 2b. However, the magnitude of the fluorescence also increased resulting in the saturation limit of the CCD detector been reached. The saturation limit of this device is shown by the flat line of the spectra as it approaches 60,000 counts in intensity during the hours of 91.5–97. In order to reduce the intensity below this limit, the integration time was decreased to 60 s and the number of averages was increased to 27 as shown in Figure 2c. A decrease in the intensity of the spectra was observed, however the weak Raman signals were effectively masked out by the remaining broad background fluorescence signal. Therefore the remaining spectra collected for this fermentation was effectively unusable (Figure 2d) as no quantitative information could be extracted due to the dominance of the broad fluorescence signal. The spectra collected using the 993 nm Raman device was only affected by moderate fluorescence as shown in Figure 3a where clear Raman peaks are visible throughout the entire batch. As a result, the tuning parameters of the device were kept constant throughout the fermentation.

### 3.2. Glucose Predictions of 903 and 993 nm Raman Spectroscopic Devices

**Figure 4:**
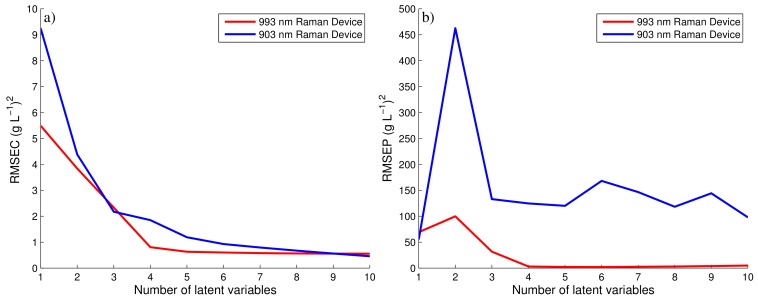
RMSEC and RMSEP for 993 and 903 nm Raman device.

The spectral data of each device and the corresponding off-line glucose measurements were used to generate two separate PLS models as previously discussed. The number of components of each model were chosen based on the RMSEC and RMSEP of both PLS models as defined by Equation (6). Figure 4 compares these errors against the number of components of each PLS model for both the 903 and 993 nm Raman devices. For the 993 nm Raman device, four components were selected as it corresponded to the lowest RMSEP. Taking additional components unnecessarily increases the complexity of the model for a marginal decrease in the error of both the RMSEC and the RMSEP. Similarly for 903 nm Raman device, three components were chosen based on similar observations. The RMSEP for 903 nm Raman device is very poor in comparison to the 993 nm Raman device suggesting the choice of components will have little influence on the predictability of the PLS model. The generation of this PLS model is summarised in Figure 3c,d, showing the off-line glucose predictions for the 993 and 903 nm Raman device, respectively. As the spectral data generated from the 993 nm device was only moderately effected by fluorescence, this device generated accurate PLS predictions when compared to the fermentation’s off-line glucose concentrations as highlighted in Figure 3c. Abu-Absi et al. [7] and Whelan et al. [33] demonstrated similar findings, both highlighting the ability of a 785 nm Raman probe to predict the glucose concentration on-line throughout mammalian fed-batch cell cultivations with both predictions shown to agree with the off-line measurements of the glucose concentration.

The PLS model predictions of the glucose concentration in Fermentation B using the spectra collected using the 903 nm Raman device were very poor when compared to the off-line values as shown in Figure 3d. These poor predictions are related to the observed increase in fluorescence with batch progression. Clearly the important Raman peaks related to changes in both the glucose and product concentrations are masked out by this fluorescence accounting for the poor predictions generated using the PLS model of this spectra. Additionally the large deviations in the predicted glucose concentration can be accounted for by considering the changes to the integration times and number of averages shown in Table 1. As the manipulation of these parameters are not accounted for in the linear relationship generated by the PLS model and can be observed by the glucose predictions shown in Figure 3d.

### 3.3. Influence of Raman Spectroscopic Incident Wavelength and Detector on Fluorescence

The two main factors contributing to the large difference observed in the intensity of fluorescence effecting the spectra collected by both Raman analysers is related to the incident wavelength of each device and the detector material used. The choice of the excitation wavelength can significantly impact the level of observed fluorescence. In Raman spectroscopy the scattered energy of the light source is inversely proportional to the fourth power of the excitation wavelength defined as:

(8) $$I_{\mathrm{Raman}}\approx {\left(\nu_{0}\right)}^{4}\approx {\left(\frac{1}{\lambda_{0}}\right)}^{4}$$

where:
$I_{\mathrm{Raman}}$ :Intensity of Raman scattered light$\nu_{0}$:Frequency of light source$\lambda_{0}$:Wavelength of light source

Therefore the longer wavelength of 993 nm Raman device results in a decrease in energy of the light source compared to the 903 nm, hence reducing the probability of fluorescence by lowering the energy available to excite the electrons of the sample up to their quantum states. Frank et al. [34] also highlighted the importance of incident wavelength selection by studying the Raman spectra collected from a human breast biopsy sample using seven different excitation wavelengths ranging from 406 nm to 830 nm. The spectra collected with the 406 nm incident wavelength was completely dominated by a broad fluorescence peak whereas the spectra collected using the higher incident wavelengths (784 and 830 nm) resulted in high resolution spectra that enabled quantitative information to be extracted from the spectral sample. Similarly, Volodin et al. [35] also demonstrated comparable results highlighting the ability of a 1064 nm Raman device to correctly characterise a dark rum sample containing a strong fluorescence background. However, when analysing the same sample using a 785 nm Raman device, the rum sample could not be characterised as the signal was corrupted by high fluorescence. Similar to the results presented here, the 785 nm Raman device used a CCD detector and the 1064 nm Raman device had an InGaAs detector in their work.

Furthermore, the 903 nm Raman device uses a CCD detector which was highlighted to have low quantum efficiency above wavelengths greater than 800 nm [15,36]. Li et al. [37] demonstrated a similar rapid decrease in the quantum efficiency of CCD detectors in wavelengths above 850 nm. In these regions the photon energy decreases below the silicon bandgap energy and the CCD detector becomes transparent to the incident photons. This reduction in quantum efficiency combined with the strong background fluorescence signal reduces the ability of this 903 nm device to detect the weak Raman peaks. McCreery [2] also highlights the significant drop in quantum efficiency of CCD detectors above 850 nm and highlights the optimum incident wavelength of Raman devices with CCD detectors to be in the range of 600–850 nm. In contrast, however, Adar et al. [36] demonstrated that the indium gallium arsenide (InGaAs) detector arrays have a high quantum efficiency at these higher wavelengths which is demonstrated by the ability of the 993 nm device to produce clearly defined Raman peaks throughout the entire fermentation. Figure 3 highlights the importance of correct detector material in addition to incident wavelength when selecting a Raman spectroscopy device for a highly fluorescence fermentation. However, it must be noted that fluorescence is sample and process specific. Raman devices using low wavelength excitation sources can be implemented successfully for samples effected by low or moderate fluorescence.

### 3.4. API Predictions of 993 nm Raman Spectroscopic Device

**Figure 5:**
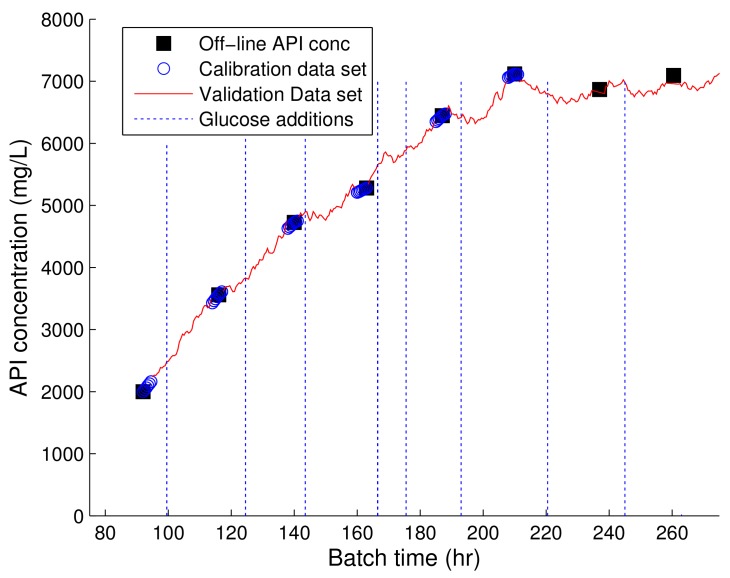
Profile of PLS model of product generated using the spectra collected from the 993 nm Raman device and the corresponding off-line concentrations of the API concentration. The dashed vertical lines represent the time of the glucose additions.

The on-line prediction of the API concentration of this fermentation was also investigated. The PLS predictions of the API concentration in comparison to the off-line values using the spectra collected from the 993 nm Raman device is shown in Figure 5. The product concentration predictions are in good agreement with the off-line measurements. The ability to estimate the product concentration on-line allows for the development of improved control strategies capable of improving product yields. The prediction of the product in Fermentation B using the 903 nm Raman were very poor and as a result these predictions are not shown. To date, few examples have reported on the ability of Raman spectroscopy to accurately model the API concentration in fermentations processes. Examples include, Cannizzaro et al. [31] who demonstrated the ability of 785 nm Raman device for the on-line production of carotenoid production in a fed-batch *P. rhodozyma* fermentation in addition to the prediction of antibody product concentrations in mammalian cell cultures [12,38]. The ability of the 993 nm Raman device to accurately predict the API concentration in this fermentation system highlights the potential benefits of applying this technology for the implementation of Quality by Design methodologies for fermentation process improvements.

## 4. Conclusions

Fluorescence is a major problem experienced by many scientists and engineers implementing Raman spectroscopy to monitor and control biopharmaceutical processes. This paper is the first direct comparison of two different Raman spectroscopy devices on the same fermentation highlighting the significant influence of incident wavelength and detector material on fluorescence levels detected by each device. The spectra recorded by the Raman spectroscopy device with the 903 nm incident wavelength and a CCD detector was corrupted by high fluorescence and rendered the recorded spectra unusable for regression analysis. However, the spectra recorded by the Raman spectroscopy device with the 993 incident wavelength and an indium gallium arsenide (InGaAs) detector generated spectra with only moderate levels of fluorescence. The spectra recorded by this device enabled accurate estimations of both glucose and API concentrations through the generation of a PLS regression model. Therefore this work demonstrates that although a lower incident wavelength increases the Raman scattering effect it can also increase the level of fluorescence rendering the recorded spectra obsolete. However, at elevated incident wavelengths the probability of fluorescence is significantly reduced in addition to the Raman scattering effect which can be compensated for by a more sensitive detector material as demonstrated by the 993 nm Raman probe with the InGaAs detector. Thus Raman spectroscopy is a highly suitable tool for the quantification of the key process parameters in biopharmaceutical processing. However, caution is advised in implementing this novel tool particularly in the choice of the appropriate incident wavelength of the analyser and the sensor detector material to ensure problems relating to high fluorescence do not impact on the quality of the recorded spectra.

## Figures and Tables

**Table 1:** Summary of tuning parameters chosen for the 993 and 903 nm Raman throughout each batch.

Spectra Reference	Start (h)	End (h)	# of Spec	Integration Time (s)	# of Averages	Issues Encountered
	993 nm Raman Device					
Spec${}_{0-260}$	0	260	520	180	9	Moderate Fluorescence
	903 nm Raman Device					
Spec${}_{0-90.5}$	0	90.5	181	180	9	Moderate Fluorescence
Spec${}_{91.5-97}$	91.5	97	10	270	6	CCD saturated
Spec${}_{98.5-114.5}$	98.5	114.5	32	60	27	High Fluorescence
Spec${}_{115.5-146.5}$	115.5	146.5	62	90	18	High Fluorescence
Spec${}_{146.5-193}$	146.5	193	93	60	27	High Fluorescence
Spec${}_{193.5-239}$	193.5	239	91	30	54	High Fluorescence

**Figure A2:**
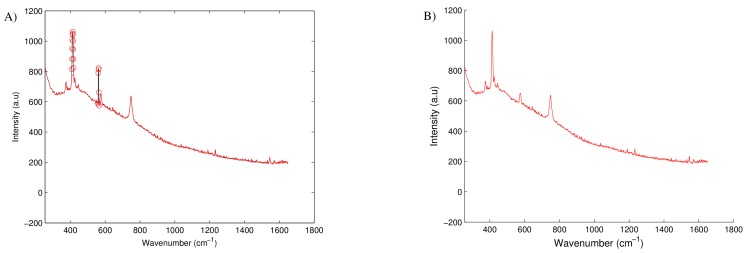
Example of spectra containing a cosmic ray with (**A**) highlighting the outlier peaks identified by the de-spiking function and (**B**) highlighting the final spectra with the cosmic ray removed. The cosmic ray located at 560 cm${}^{-1}$ is correctly identified as an outlier, however this function also wrongly identified the strong sapphire peak located at 410 cm${}^{-1}$ as an outlier. This demonstrates the importance of viewing each of the identified outliers before they are blindly removed.

## Abbreviations

The following abbreviations are used in this manuscript:

API	Active pharmaceutical ingredient
B	Inner-relationship matrix for PLS model
CCD	Charged couple device
CPP	Critical process parameters
CQA	Critical quality attributes
E	Residual matrix in PLS model for X-data
F	Residual matrix in PLS model for Y-data
FDA	Food and drug administration
HPLC	High pressure liquid chromatography
InGaAs	Indium gallium arsenide
*n*	Number of calibration points in PLS model
NIPLAS	Non-linear iterative partial least squares
*p*	Number of validation points in PLS model
P	Loadings matrix in PLS model
PAT	Process analytic technology
PID	Proportional integral derivative
PLS	Partial least squares
*R*	Number of latent variables in PLS model
RMSEC	Root mean square error of calibration
RMSEP	Root mean square error of prediction
UV	Ultra-violet
U	Scores matrix in PLS model of Y-data
Q	Loadings matrix in PLS model of Y-data
T	Scores matrix in PLS model of X-data
$X_{spec}$	X-data (spectral) in PLS model
$y_{i}$	*i*th calibration point in PLS model
${\hat{y}}_{i}$	*i*th validation point in PLS model
$Y_{Gluc}$	Y-data (glucose) in PLS model
$Y_{API}$	Y-data (API) in PLS model
β	Regression coefficients for PLS model
$\nu_{0}$	Incident wavelength of Raman device
*h*	Planks constant

## Appendix A. Raman Spectroscopy Operation

During the operation of Raman spectroscopy devices there are two main tuning parameters that can be manipulated, these parameters influence the quality of the spectra collected, where:

- **Integration time**: relates to the detector exposure time, the longer the integration time the larger the intensity of the Raman spectra. The intensity relates to the total accumulated charge recorded on a single pixel. Large integration times can saturate the detector whereas small integration times can decrease the Raman peaks below detectable levels, therefore a balance is required.
- **Number of averages**: refers to the number of spectra that were averaged to obtain a single spectrum, used to improve the signal to noise (SNR) ratio.

### Appendix Overview of Spectral Preprocessing Methods

Similar to the majority of spectroscopic analysis, Raman spectra must be preprocessed to improve the predictability of the classification and regression models generated. The objective of preprocessing is to remove all the unwanted physical phenomena that are uncorrelated with the process. Bocklitz et al. [6] regards the following as the three primary disturbances associated with Raman spectroscopy,

1. **Fluorescence and background Baseline Increase**
2. **Noise**
3. **Cosmic spikes**

These unwanted artifacts are discussed below, highlighting the primary preprocessing methods employed to remove or diminish their effects.

**1. Fluorescence and background baseline increase**

Fluorescence as discussed is a major problem in Raman spectroscopy often highlighted by a broad background signal and/or baseline increase across the recorded spectra. Provided this fluorescence does not completely inundate the weak Raman emission of the material of interest, preprocessing of the spectra can remove this moderate fluorescence and help generate improved regression models. There are various different preprocessing techniques available with polynomial fitting or calculating the derivative of the spectral signal as the two most widely accepted techniques. Polynomial fitting involves the subtraction of a least-squares fitted polynomial from the spectra. A fourth- to sixth- order polynomial is generally used to approximate the fluorescence background signal and is then subtracted from the original Raman spectra. This technique, however, can result in negative baseline corrected spectral values which are difficult to interpret and non-physical [39]. An extension of this technique is to perform weighted least-squares baseline correction which performs an optimisation function across the whole spectra. Points with residuals greater than zero are weighted at each iteration of the least-squares fitting algorithm resulting in non-negative baseline corrected spectra. The application of this technique to some calibration spectra collected is shown in Figure A1, with further details of the algorithm found in [24].

Alternatively the baseline shift can be corrected for by working with the first or second derivative of a spectra. This transformation is linear with the curves produced retaining their quantitative aspects in respect to the original spectra [26]. However, derivative calculations can sometimes amplify spectral noise. This is avoided by smoothing the data before calculating the derivative. A Savitzky-Golay filtering technique is often the smoothing technique of choice for Raman spectroscopic analysis, this technique fits a *n*th order polynomial to *k* input samples iteratively smoothing the spectral data set [40].

**2. Noise**

Noise is an inherent disturbance of any sensor, generally for Raman spectroscopy this noise can be a result of thermal noise, instrument read out noise or even as a result of cosmic rays. In Raman spectroscopy the noise is usually characterised by its high frequency. To minimise its effect, the noise can be reduced or eliminated through the application of a filtering or smoothing technique.

**3. Cosmic Peaks**

Sharp, intense peaks are often the result of random high energy particles (i.e., cosmic rays) hitting the detector. Although these artifacts are a rare occurrence they are generally easy to detect and obvious through visual inspection of the spectra. It is necessary to remove these peaks as they may interfere with spectral analysis particularly during data smoothing or scaling. To remove these peaks, a de-spiking function can be applied. The de-spiking function applied in this work is detailed in Mori et al. [23] with its application demonstrated in Figure A1. The method calculates the average angle of every peak along the spectra and determines if any of the peaks are abnormal large (i.e., a cosmic peak). This abnormal peak is identified and subsequently removed and replaced through data interpolation.
